# Pharmacogenetics of Drugs Used in the Treatment of Cancers

**DOI:** 10.3390/genes13020311

**Published:** 2022-02-07

**Authors:** Beata Franczyk, Jacek Rysz, Anna Gluba-Brzózka

**Affiliations:** Department of Nephrology, Hypertension and Family Medicine, Medical University of Lodz, Zeromskiego 113, 90-549 Lodz, Poland; beata.franczyk-skora@umed.lodz.pl (B.F.); jacek.rysz@umed.lodz.pl (J.R.)

**Keywords:** pharmacogenetics, SNPs, poor metabolizer, ultra-rapid metabolizer

## Abstract

Pharmacogenomics is based on the understanding of the individual differences in drug use, the response to drug therapy (efficacy and toxicity), and the mechanisms underlying variable drug responses. The identification of DNA variants which markedly contribute to inter-individual variations in drug responses would improve the efficacy of treatments and decrease the rate of the adverse side effects of drugs. This review focuses only on the impact of polymorphisms within drug-metabolizing enzymes on drug responses. Anticancer drugs usually have a very narrow therapeutic index; therefore, it is very important to use appropriate doses in order to achieve the maximum benefits without putting the patient at risk of life-threatening toxicities. However, the adjustment of the appropriate dose is not so easy, due to the inheritance of specific polymorphisms in the genes encoding the target proteins and drug-metabolizing enzymes. This review presents just a few examples of such polymorphisms and their impact on the response to therapy.

## 1. Introduction

Pharmacogenomics is based on the understanding of individual differences in drug use, the response to drug therapy (efficacy and toxicity), and the mechanisms underlying variable drug response with the use of genomics, proteomics, transcriptomics, and metabolomics [1,2]. Genome sequencing and mutations analysis are crucial tools in pharmacogenomics [3]. Pharmacogenomics involves the study of both the patient’s and the tumour’s genome, as variations in both have been observed to exert an impact on the transport, efflux, retention, and penetration of the anti-cancer drug [3]. We all have a different genetic makeup; therefore, we respond in different ways to drugs and environmental factors, and we have a diverse risk of developing diseases [4]. Variations in the human genome occur approximately every 300–1000 nucleotides, and there are more than 14 million single nucleotide polymorphisms (SNPs) spread throughout the entire human genome [4]. The rapid development of technology enabled a better understanding of the differences between individuals in terms of one or more traits, behaviours, or characteristics. One group of patients administered with the same drug in the same dose may respond well to the treatment, in another group the treatment would not elicit adequate responses, while in the third group the drug will cause serious side-effects or even death. Therefore, the identification of the DNA variants which markedly contribute to inter-individual variations in drug responses would improve the efficacy of treatments and decrease the rate of the adverse side effects of drugs. Numerous studies have indicated that there are many causative factors which are responsible for variations in drug response, and which exert a direct or indirect impact [2]. Apart from inherited genetic factors, environmental factors (exposure to radiation and some chemicals), lifestyle factors (smoking, drinking, and exercise) and physiological factors (age, sex, kidney and liver function, and pregnancy) may be of importance [5]. Currently, it is known that the drug response depends on the pharmacokinetic and pharmacodynamic properties of the prescribed drugs and the individual patient’s polymorphisms within drug-metabolizing enzymes and transporters [2]. Increasing evidence suggests that such variants can directly impact the function of drug-metabolizing enzymes and transporters, which translate into altered efficacy and/or the occurrence of adverse drug reactions (ADRs) [1]. Owing to growing scientific data, the United States Food and Drug Administration (FDA) and the European Medicines Agency (EMA) listed over 120 drugs for which genomic biomarkers should be determined in order to adjust the dosing and assess the safety risk or efficacy [6,7,8]. Among the FDA-approved biomarkers for anti-cancer drugs, there are: cytochrome P450 2D6 (CYP2D6) for tamoxifen; rucaparib and dihydropyrimidine dehydrogenase (DPYD/DPD) for fluorouracil and capecitabine; and thiopurine S-methyltransferase (TPMT) for cisplatin, mercaptopurine, and thioguanine.

## 2. Variations in Individual Drug Response

As we mentioned above, individuals vary considerably in their clinical responses to administered drugs and their outcomes [2]. Such inter-individual dissimilarities frequently pose a challenge to the optimization of a dosage regimen because, according to estimations, most drugs are efficacious in only 25–60% of patients [9]. Many patients do not fully respond to and benefit from the initial recommended drug treatment. Even as many as 75% of patients who have cancer show no response to the first therapy [10]. The response to the same drug and dose can also be different in various patients. The same dose may be ineffective in a group of patients due to a too-low drug concentration, while in other group it can lead to the occurrence of serious side effects or even be lethal [2]. In order to avoid unexpected and undesirable outcomes, patients taking drugs with narrow therapeutic indexes should be carefully monitored [11]. The situation worsens if the patient is also administered other drugs as a result of possible drug–drug interactions. The presence of comorbidities can lead to the occurrence of adverse drug–disease interactions [12]. Based on twin studies aiming to analyse the genetic component contributing to response variation, Kalow et al. [13] hypothesized that different genetic factors determined approximately 20–95% of the inter-individual variability in drug responses. Moreover, it was found that dizygotic twins displayed more metabolic variability than monozygotic twins for isoniazid metabolism [14]. Furthermore, the metabolism of antipyrine, halothane and phenytoin was demonstrated to be associated with genetic factors and exposure to a disease-favouring environment [15,16]. Whilst the individual differences in responses related to genetic factors are mostly permanent, those associated with other factors are frequently transient [17]. This is in agreement with the observation of Vesell et al. [18], who found a relatively higher variability of a drug response in the population compared to the intra-individual response variability at different times. This review focuses only on the impact of polymorphisms within drug-metabolizing enzymes on drug response. In general, drug-metabolizing enzymes can be divided into two main categories: phase I metabolizing enzymes, which are involved in the addition or removal of functional groups via reduction, oxidation or hydrolysis, and phase II metabolizing enzymes, which are responsible for the transfer of moieties from a cofactor to a substrate [19]. Polymorphisms within the genes of phase-1 drug-metabolizing enzymes or transporters can alter drugs’ pharmacokinetics (drug absorption, distribution, metabolism, and excretion), pharmacodynamics (the interaction of drugs with targets, including enzymes, receptors and ion channels), or both, resulting in differences in drug responses [20,21,22]. Enzymes from both categories display genetic polymorphisms, some of which may prove clinically relevant. Phase I metabolizing enzymes participate in the reduction of nicotinamide adenine dinucleotide phosphate (NADPH)-cytochrome P450 reductase and reduced cytochrome P450, the oxidation of cytochrome P450, dihydropyrimidine dehydrogenase, aldehyde dehydrogenase, alcohol dehydrogenase, flavin-containing monooxygenase, and monoamine oxidase, as well as the hydrolysis of amidases, epoxide hydrolase and esterases [19]. Drug-metabolizing CYPs are mostly located in the liver and intestinal mucosa [23]. It appears that variations in the cytochrome P450 (CYP) superfamily exert the most important clinical effect, as these enzymes not only take part in the transformation of most of the clinically used drugs (90%) but also toxins and carcinogens [19]. It is estimated that CYPs 1A2, 2B6, 2C8, 2C9, 2C19, 2D6, 3A4, and 3A5 are involved in the metabolism of the top 200 prescribed drugs in the USA [24]. The results of studies have indicated that human CYP genes are highly polymorphic, and these mutations can result in the complete abolition, reduction, lack of effect or enhancement of enzyme activity [19]. For example, inter-individual differences in CYP1A2 expression and activity in the human liver range from 10- to 200-fold, while hepatic CYP2B6 expression varies 250-fold among individuals [23,25,26]. CYP2D6, CYP2C19, and CYP2C9 are the most common CYP enzymes which are susceptible to polymorphisms. The following four phenotypes have been determined based on CYP enzyme activity: poor metabolizers (activity is abolished), intermediate metabolizers with decreased enzyme activity, extensive metabolizers characterized by normal activity, and finally ultrarapid metabolizers with increased activity [19]. Poor metabolizers would display higher concentrations of a drug which is metabolized by a polymorphic enzyme, and such a concentration would be maintained for a longer time; therefore, it appears that such patients should be administered lower doses in order to avoid the occurrence of adverse reactions. In turn, ultrarapid metabolizers should be treated with higher doses of a drug in order to reach optimal efficacy, as in this case the drug is metabolized too fast, and sometimes its therapeutic concentration in plasma is not obtained. The opposite situation is observed when the patients is administered a prodrug which must undergo metabolic activation. In this situation, poor metabolizers may not respond to treatment due to a low drug concentration, while ultrarapid metabolizers may face drug toxicity. Uridine diphosphate glucuronosyltransferase (UGT), glutathione S-transferases (GST), sulfotransferase (SULT), N-acetyltransferase (NAT) and thiopurine methyltransferase (TPMT) are the most significant phase II enzymes [27]. Genetic polymorphisms within the *UGT* gene were found to be able to modify the function or expression of the protein, possibly changing the enzyme glucuronidation capacity. As a result of multiple GST biological activities, functional polymorphisms within its gene may alter the cancer susceptibility, therapeutic response, and prognosis [28,29].

## 3. The Impact of Genetic Variations on Anticancer Treatment Efficiency

### 3.1. 5-Fluorouracil (5-FU)

Today, 5-fluorouracil remains one of the most frequently prescribed anticancer drugs for the therapy of gastrointestinal tract malignancies (e.g., colorectal and gastric cancer) [19]. This drug has a narrow therapeutic window, which means that there is a small difference between the minimum efficacious and maximum tolerable dose [30]. Approximately 80% of the administered dose is degraded and subsequently excreted in the urine, while 1–3% of the 5-fluorouracil is metabolized to cytotoxic metabolites. The 5-FU is administered in the form of a prodrug (fluoropyrimidine analogue) which is converted to the active metabolite (5-fluoro-2-deoxyuridine monophosphate) (FdUMP) via three pathways: oratate phosphoribosyltransferase (OPRT), uridine phosphorylase (UP), and thymidine phosphorylase (TP). The active drug inhibits thymidylate synthase (TS), which results in the inhibition of DNA synthesis [31]. The 5-fluorouracil is mainly (~80–85%) metabolized by the enzyme dihydropyrimidine dehydrogenase (DPD) in the liver; this is a rate-limiting enzyme [3]. Following the transformation to the inactive form (dihydrofluorouracil) (FDHU), it is excreted in the form of a fluoro-β-alanine. The results of studies have demonstrated that the expression of DPD is associated with the tolerance and the response to 5-FU-based chemotherapy [19]. Roughly 10–40% of fluoropyrimidine-treated patients suffer from severe and sometimes life-threatening toxicity, including vomiting, nausea, severe diarrhoea, mucositis, stomatitis, and neutropenia hand–foot syndrome [32,33,34]. Variations in the expression and levels of DPD are related to the presence of genetic aberrations. According to estimations, 3–5% of the population is partly or totally deficient in DPD enzyme activity [35]. Some polymorphisms in the *DPD* gene have been confirmed to prolong the half-life of drugs, which results in substantial toxicity, including myelosuppression, mucositis, and hand–foot syndrome, etc. [3]. According to studies, four *DPYD* variants are of principal importance, taking into consideration their population frequency and recognised impact on enzyme function and toxicity risk: c.1905+1G>A (rs3918290, also known as DPYD*2A, *DPYD:IVS14+1G/A*), c.1679T/G (rs55886062, DPYD*13, p.I560S), c.2846A/T (rs67376798, p.D949V), and c.1129–5923C/G (rs75017182, *HapB3*) [30]. In particular, c.1905+1G/A and c.1679T/G exert the greatest deleterious impact on DPD activity; in turn, the impact of c.2846A/T and c.1129–5923C/G is moderate. Low levels of this enzyme translate into the accumulation of 5-FU, and a subsequent increased risk of severe toxicities [36]. The absence of the enzyme can even result in lethal toxicities when receiving 5-FU-based chemotherapy. In turn, the high expression of DPD leads to a poor response to the treatment [37]. Morel et al. [38] stated that three SNPs (IVS14+1G/A, 2846A/T, 1679T/G) were associated with grade 3 to 4 toxicity. Patients who are homozygous for the IVS14+1G/A allele are completely lacking DPD activity; therefore, 5-FU-related toxicities can be life-threatening, or sometimes even fatal [39,40]. The authors suggested the pre-treatment analysis of three *DPYD* SNPs in order to avoid severe toxic side effects. Moreover, they stated that even in the case of dihydropyrimidine dehydrogenase deficiency, 5-FU can be safely administered, but an individual dose must be determined. Another study of solid-tumour patients demonstrated that SNPs c.1905G/A (rs3918290; IVS14+1 G/A), c.1679T/G (rs55886062), and c.2846A/T (rs67376798)—with frequencies of 3%, 0.3%, and 26%, respectively—were associated with toxic effects, i.e., grade 3 and 4 toxicities on treatment with 5-FU [41]. A meta-analysis of data from eight cohort studies (*n* = 7365 patients) revealed that the relative risks for toxicity in the case of the polymorphisms c.1905+1G/A (*2A), c.2846A/T, c.1679T/G (*13), and c.1129–5923C/G (HapB3) were 2.9 (95%CI: 1.8–4.6), 3.0 (2.2–4.1), 4.4 (2.1–9.3), and 1.6 (1.3–2.0), respectively [32]. Nie et al. [42] also demonstrated that the presence of c.1905+1G/A and c.1679T/G translated to a 50% and 68% reduction in heterozygous carriers, respectively, while c.2846A/T and c.1129–5923C/G were associated with a 30% and 35% decrease in activity, respectively, in heterozygous carriers. In turn, other studies found that the homozygous expression of c.1905+1G/A and c.1679T/G resulted in severely diminished DPD activity below 25% of wild-type activity, while in case of c.2846A/T the DPD activity was 39–59% of the wild-type activity [43,44]. The appropriate dose for patients with polymorphisms within DPD can be adjusted on the basis of the calculated DPD activity score (DPD-AS). However, such recommendations take into consideration only the known SNP, and therefore it seems that, in some cases, it may be not ideal. DPD-homozygous patients with reduced or abolished DPD activity should be administered with a decreased starting dose. Deenen et al. [45] demonstrated that the 50% reduction of dose in heterozygous carriers of no-function variant c.1905+1G/A enabled the reduction of severe toxicity to levels comparable to non-carriers. However, there is only limited evidence on the optimum degree of dose reduction in carriers of decreased-function variants. Due to the fact that some carriers of variants with reduced or no activity tolerate the standard dose of 5-FU, the drug doses should be increased in successive cycles in order to preserve efficiency in those experiencing no or clinically acceptable toxicity during the first cycles of chemotherapy [30]. On the other hand, the doses should be reduced in patients who cannot tolerate the starting dose. In general, the avoidance of 5-fluorouracil-containing regimens is recommended in DPD poor metabolizers, unless no other fluoropyrimidine-free regimens are considered. At that time the initial drug dose should be considerably reduced and therapeutic monitoring ought to be early implemented. However, no evidence of successful treatment with 5-FU in DPD poor metabolizers has been reported so far. It has been suggested that the starting dose of 5-FU in patients with reduced or no DPD function should be no higher than 25% of the normal dose. The warning concerning the use of 5-fluorouracil in DPD-deficient patients has been added to the drug label by the US Food and Drug Administration (FDA) and the Health Candida Sante Canada (HCSC) [30].

Indeed, a pharmacokinetically-based dosing of 5-fluorouracil has been found to bring about effects because it increases the amount of patients treated with the optimal dose, and decreases 5-FU’s adverse effects [46,47].

A further stratification of DPD risk variant carriers will possibly by owing to the identification of a polymorphism (rs895819 A/G) in the DPYD-regulatory microRNA miR-27a, which is associated with lower DPD activity [48]. However, no combined pharmacokinetic studies are available; therefore, dosing recommendations cannot be made on the basis of the MIR27A genotype. Moreover, it should be kept in mind that apart from genetic variations, patient characteristics—including age, sex, disease and treatment regimens—have also been associated with 5-fluorouracil toxicity [30].

What is interesting is that homozygous carriers of inactivating variants of DPYD not only have problems with the metabolism of fluorouracil and capecitabine but also suffer from complete dihydropyrimidine dehydrogenase deficiency, which is a clinically heterogeneous autosomal recessive disorder of pyrimidine metabolism characterized by a variety of clinical presentations, even involving severe convulsive disorders with motor and mental retardation [49,50].

The individual efficacy of 5-FU- based therapy may also depend on genetic variations within molecules which are responsible for the transport of this drug and/or activated compounds [51]. The ATP-binding cassette subfamily G member 4 (ABCC4) participates in the transport of numerous endogenous and exogenous organic anions out to the cell [51]. This protein was found to confer cells with a resistance to cytotoxic complexes, prevent the aberrant biological damage of vital tissues, and exert an impact on drug metabolism in cells, leading to drug resistance. Chen et al. [51] reported the impact of rs3742106 polymorphism in the 3′-UTR of the *ABCC4* (multi-drug resistance-associated protein 4) gene on the efficacy of 5-FU and capecitabine-based chemotherapy in colorectal cancer (CRC). The carriers of the rs3742106 T/T genotype turned out to be much more sensitive to the therapy of 5-FU compared with G/G genotype carriers. The presence of the T allele is associated with the formation of a binding-site for miR-3190-5p which results in reduced ABCC4 protein expression and higher levels of intracellular 5-FU, as well as the enhanced sensitivity of CRC cells to 5-FU chemotherapy [51]. The results of studies have suggested that the miRNAs miR-124a, miR-125a, miR-125b, miR-143, and miR-506 may attenuate the expression of variant ABCC4 [52,53]. Indeed, miR-3190-5p was demonstrated to directly recognize the rs3742106 T-allelic 3′-UTR of *ABCC4*, and to reduce its expression in a dose-dependent manner [51]. In turn, increased ABCC4 expression was observed in 5-FU-resistant cells [54]. Apart from ABCC4, genetic variations in *ABCG2*—i.e., C421A—could also affect the protein expression and individual efficacy of antineoplastic drugs resulting from the regulatory role of hsa-miR-519c and hsa-miR-32816.

### 3.2. Irinotecan

Irinotecan (a semisynthetic analogue of camptothecin) is the topoisomerase I inhibitor. This drug exerts a potent antitumor activity against a wide range of tumours; as such, it is one of the most commonly administered chemotherapy agents [55]. It is widely used in the treatment of metastatic colorectal cancer, either in combination with 5-fluorouracil as the first-line therapy, or as a monotherapy in the second-line treatment [56]. Irinotecan is a prodrug which is transformed by human carboxylesterase 1 and 2 (hCE1 and hCE2) into active 7-ethyl-10-hydroxycamptothecin (SN-38). SN-38 has been shown to be 100- to 1000-fold more cytotoxic than irinotecan [57]. SN-38 is then detoxified by UDP glucuronosyltransferase family 1 member A1 (UGT1A1) (the glucuronidation of SN-38) to form the less toxic, inactive metabolite/β-glucuronide derivative of SN-38G, which is excreted into the bile [58]. Apart from UGT1A1, irinotecan also undergoes deactivation via CYP3A4-mediated oxidation to form two inactive metabolites: 7-ethyl-10-(4-N-(5-aminopentanoic acid)-1-piperidino) carbonyloxycamptothecin (APC) and 7-ethyl-10-(4-amino-1-piperidino) carbonyloxycamptothecin (NPC) [59,60]. CYP3A4 is expressed in intestinal enterocytes; therefore, it contributes significantly to the first-pass metabolism of orally administered drugs [61,62]. The observed inter-individual variability in hepatic CYP3A4 expression is very high (>100-fold); however, the presence of none of the already-identified 43 variant alleles cannot explain this variability [23,63]. The dose-limiting toxicities of irinotecan (diarrhoea and myelosuppression) restrict the optimal utilization of this drug. Such toxicities of irinotecan are frequently related to higher SN-38 levels. The observed significant interpatient variability in SN38G formation suggested the crucial role of genetic variations in *UGT1A1* [64]. Genetic polymorphisms in the metabolic enzymes and transporters involved in irinotecan disposition are partly responsible for the considerable variability in its pharmacokinetics, efficacy, and toxicity profiles [65]. Patients who are genetically predisposed to reduced UGT1 activity (e.g., in Gilbert’s syndrome) show a higher susceptibility to the development of severe toxicity when treated with irinotecan [66]. The results of studies have indicated that UGT1A1*6 and *28 polymorphisms lead to increased systemic exposure to irinotecan and SN-38 in homozygous patients, thus enhancing the risk of irinotecan-associated adverse events [67,68]. The polymorphic (TA)n repeat in *UGT1A1* affects its basal transcription [69]. The prevalence of UGT1A1*28 alleles differs considerably among different ethnic groups, even reaching 35% in Caucasians and African Americans [69,70]. Moreover, Innocenti et al. [70] suggested that the haplotype structure of the promoter may be different between Caucasians and African-Americans because, in African-Americans, only marginal levels of significance were found between (TA)n and −3279 (*p* = 0.02), and between −3279 and −3156 (*p* = 0.04). The results of studies have demonstrated that the genetic variant UGT1A1*28 (an extra TA repeat in the TATA promoter sequence of the *UGT1A1*) translates to diminished SN-38 glucuronidation and a subsequent increased susceptibility to irinotecan-induced gastrointestinal and haematological toxicity [71,72,73]. Patients who are homozygous for this variant (UGT1A1*28/*28) show decreased enzymatic activity, and are predisposed to the development of myelosuppression and severe diarrhoea when treated with irinotecan [74,75]. Patients who are homozygous for the UGT1A1*28 allele should be administered with a reduced initial dose. Prospective studies have revealed that the recommended dose of 180  mg/m^2^ of irinotecan in the FOLFIRI regimen appears to be markedly lower than the dose tolerated by non-UGT1A1*28/*28 mCRC patients [76]. Clinical evidence shows that premature drug suspension and dose reduction due to toxicity can reduce antitumour activity. The results of studies have indicated that this common (TA)n polymorphism is in linkage disequilibrium with other polymorphisms within the promoter region: −3279 and −3156 (*p* < 0.0001) [70]. In Caucasians, these two promoter variants (UGT1A1 −3263T/G and −3156G/A) were found to increase the prevalence of irinotecan-induced grade 4 neutropenia or diarrhoea [71,77]. Irinotecan-related toxicity is also related to the presence of other polymorphisms in the UGT1A1. For example, in the East Asian population, UGT1A1*6 (Gly71Arg) polymorphism reducing UGT1A1 catalytic activity by 60% in homozygotes is quite frequent (~12%) [78]. In a phase I study of Korean patients genotyped for *UGT1A1*, the recommended doses were 300 for participants with no defective alleles (DA), 270 for those with one DA, and 150 for those with two DA mg/m^2^ [79]. Two other retrospective studies involving Asian patients confirmed that patients with mCRC who had an initial dose of irinotecan adjusted on this basis could achieve a more favourable response and outcome without a considerable rise in toxicity (with a FOLFIRI-plus-bevacizumab regimen) [76,80]. The presence of at least one UGT1A1*6 allele has been demonstrated to increase the risk of neutropenia and diarrhoea in Asian patients with gastrointestinal tumours or NSCLC; however, this relationship appeared not to be dose-dependent [81,82]. However, these two SNPs appear not to influence the tumour response to the treatment in Asian NSCLC or SCLC patients receiving irinotecan as first- or second-line chemotherapy [81]. In turn, UGT1A1*93 (rs10929302; −3156G/A)—which is in linkage disequilibrium with UGT1A1*28 and decreases *UGT1A1* expression—was found to be associated with elevated bilirubin concentrations in homozygous patients [71]. Moreover, this variant increased SN-38 AUC and the incidence of hematologic toxicities (including neutropenia), diarrhoea, grade 3 vomiting, and a diminished neutrophil count [83,84,85]. The carriers of UGT1A7*3 and UGT1A7*4 polymorphisms have reduced enzyme activity and SN-38 conjugation, which translate into a greater risk of adverse events during irinotecan chemotherapy [86,87,88]. *UGT1A1* genotyping should be performed in order to avoid the unnecessary risk of serious side effects. It appears that patients with UGT1A1*1/*28 and UGT1A1*28/*28 alleles would benefit from the reduction of the dose. Currently, there is no evidence showing whether such a dose reduction would influence the tumour response. The United States of America Food and Drug Administration confirmed the importance of UGT1A1 pharmacogenetics in the determination of the dose and the prediction of toxicity to irinotecan. They recommended the reduction of this drug dose in homozygous patients; however, they did not specify the exact dose reduction required to limit drug toxicity for *28/*28 patients [67]. Prospective studies have revealed that the recommended dose of 180  mg/m^2^ of irinotecan in the FOLFIRI regimen appears to be markedly lower than the dose tolerated by non-UGT1A1*28/*28 mCRC patients [89]. A further genotype-driven phase I study of irinotecan administered in combination with fluorouracil/leucovorin in patients with the same type of cancer demonstrated that 370  mg/m^2^ in the *1/*1 genotype and 310  mg/m^2^ in the *1/*28 genotype can be safely administered every 2 weeks in patients undergoing first-line treatment [90]. In turn, in a multicentre phase II ERBIFORT study, the combination of cetuximab and irinotecan 260  mg/m^2^ for UGT1A1*1/*1 and *1/*28 genotypes, and 220  mg/m^2^ for UGT1A1*28/*28 genotypes was found to yield high response rates, and enabled the complete resection of hepatic metastases in most patients with resectable liver metastases of CRC [91]. Owing to dose adaptation, the administered treatment was less toxic and effective. A clinical, randomised, phase II trial to evaluate the efficacy and safety of FOLFIRI with high-dose irinotecan (HD-FOLFIRI) in metastatic colorectal cancer patients according to their *UGT1A1* genotype confirmed the safety of this approach; it also demonstrated that despite not improving the survival, such treatment enhanced the overall response rate [92]. Still, randomized III phase trials are required to validate the benefits of irinotecan intensification according to *UGT1A1* pharmacogenetics.

Apart from metabolizing enzymes, polymorphisms within transport proteins may also affect the efficacy of irinotecan. The increased expression of ATP-binding cassette subfamily B member 1 (ABCB1, P-glycoprotein (P-gp)) participating in the biliary excretion of CPT-11 and SN-38 has been found to raise SN-38 secretion, which results in its diminished plasma levels and an enhanced risk of intestinal toxicity, decreasing at the same time the risk of neutropenia [93,94,95]. Several *ABCB1* variants—including rs1128503 (1236 C/T), rs2032582 (2677 G>T/A), and rs1045642 (3435 C/T)—have been confirmed to affect P-gp expression, SN-38 plasma concentrations, and renal clearance [96,97]. Riera et al. [93] suggested the *ABCB1* rs1128503 variant as a predictor of irinotecan-related severe gastrointestinal toxicity, especially diarrhea and mucositis. Moreover, the rs2032582 variant also seems to increase the risk of severe mucositis. Population-related pharmacogenomics revealed that the *ABCB1* (C3435T) T/T genotype was related to irinotecan-plus-cisplatin-induced diarrhea [98]. In turn, the study of genetic variations in metastatic colorectal cancer patients revealed a marked relationship between the combined presence of *ABCB1* 1236C/T, and SLCO1B1 521T/C polymorphisms, grade 3–4 toxicity, and grade 3–4 neutropenia [99]. Similar effects were obtained in the study of polymorphisms within *UGT1A1, ABCB1, ABCG2, ABCC4, ABCC5*, and *MTHFR* in patients with metastatic colorectal cancer [100]. This study demonstrated higher hematological toxicity and overall toxicity in patients carrying the polymorphisms rs1128503, rs2032582, and rs1045642 in *ABCB1* and rs1801133 in *MTHFR*. However, the correction of the p values with the use of a false discovery rate resulted in only *ABCB1* variants being statistically significant. Furthermore, Salvador-Martín et al. [100] indicated 11.3-fold and 4.6-fold higher risks of haematological toxicity (95% CI, 1.459–88.622) and overall toxicity (95% CI, 2.283–9.386) associated with *ABCB1*, respectively. They revealed that the analysis of three SNPs in *ABCB1* enabled the prediction of the overall and haematological toxicity with diagnostic odds ratios of 4.40 and 9.94, respectively. In a Swedish and Norwegian population of patients with advanced colorarctal cancer, polymorphisms in *ABCB1* translated into early toxicity and a lower response to treatment [101]. Patients with the 1236T-2677T-3435T *ABCB1* haplotype were found to be less responsive to treatment with irinotecan (43 vs 67%, *p* = 0.027), and their survival time was shorter compared to carriers of a different haplotype; OR = 1.56 (95% CI = 1.01–2.45). 

However, some other studies failed to demonstrate the relationship between these polymorphisms and irinotecan-induced severe toxicity [102,103].

### 3.3. Tamoxifen

Tamoxifen is a selective oestrogen receptor modulator which is commonly used for the treatment and prevention of ER-positive breast cancer [19]. The growth of this type of cancer usually depends on oestrogens; therefore, its treatment requires selective ER modulators which block oestrogen binding via the attachment to the ligand-binding domain of an ER. The binding of the modulator does not allow for conformational changes of ER and the subsequent binding of the co-activators. As a result, the oestrogen-driven proliferation of ER-positive tumours becomes reduced or abolished. The standard recommendation of 5 years of adjuvant therapy with tamoxifen was based on the results of the Early Breast Cancer Trialists’ Collaborative Group [104]. Adjuvant tamoxifen has been demonstrated to markedly reduce recurrence and breast cancer mortality in pre-and postmenopausal patients with primary breast cancer, within 15 years after primary diagnosis. According to the estimations, even 30–50% of patients administered tamoxifen adjuvant therapy cannot benefit from the treatment [105]. In some cases, the patient did not react to therapy, while in others cancer recurrence or adverse drug reactions (such as hot flashes, hyperhidrosis, irregular menstruation and metastatic diseases) occurred [106,107]. Tamoxifen, following administration, requires activation in order to exert its pharmacological activity. Numerous enzymes are involved in the metabolism of tamoxifen: hepatic CYP3A4, CYP3A5, CYP2C9, CYP2C19, CYP1A2, CYP2B6, and CYP2D6, and flavin-containing monooxygenase 1 and 3 (phase I), as well as SULT1A1 and UGTs (phase II) [108,109,110]; however, CYP2D6 and CYP3A4/5 play the most important role. These two enzymes transform tamoxifen into two primary metabolites—4-hydroxytamoxifen and N-desmethyltamoxifen—which are further converted (primarily by CYP2D6) into pharmacologically active 4-hydroxy-N-desmethyltamoxifen (endoxifen) [109]. This active form displays a much higher affinity for the oestrogen receptor than tamoxifen [111]. Over 145 variants of CYP2D6 have been identified, and many of them significantly modify enzyme function [23]. Functional alterations in CYP2D6 activity (as well as drug induction or drug inhibition) may affect the clinical outcome of patients treated with tamoxifen. Variations in CYP2D6 activity explain up to 39% of the variability of the plasma concentration of (Z)-endoxifen and (Z)-4-OH-tam [112]. However, the concomitant administration of some selective serotonin reuptake inhibitors (SSRIs) (e.g., paroxetine, fluoretine) or selective noradrenaline reuptake inhibitors (SNRIs) has also been found to decrease the plasma levels of endoxifen, and to adversely affect the efficacy of tamoxifen therapy [113]. Women with lower serum levels of endoxifen were found to have a higher risk of recurrence and other adverse outcomes; therefore, it appears that this is the key active metabolite of tamoxifen [114]. It has been suggested that a lower plasma endoxifen concentration may be associated with genetic variations in CYP2D6 which decrease its activity. Many studies have demonstrated a worse outcome in tamoxifen-treated patients carrying non-functional or reduced-function alleles of CYP2D6 [115,116]. The presence of two non-functional alleles of CYP2D6 in an individual confers a PM phenotype, two normally-functioning alleles represent EM phenotype, the co-occurrence of one null allele and another allele conveying diminished function gives rise to an IM phenotype, and the presence of extra normal activity *CYP2D6* gene copy/copies confers the UM phenotype [117,118,119,120]. The rate of drug metabolism via CYP2D6 in UM is much faster than that in IM or PM phenotypes, which translates into a very low plasma drug and a lack of drug efficacy [121]. Therefore, such patients require higher doses in order for the drug to be optimally effective; however, it can turn out to be life-threatening if using drugs with narrow therapeutic indexes. The frequency of the *CYP2D6* phenotype in the Caucasian population is as follows: 5–10% PMs, 10–17% IMs, 70–80% EMs, and 3–5% UMs [117]. A retrospective analysis of German and US cohorts of 1325 patients treated with adjuvant tamoxifen for early stage breast cancer revealed that poor metabolizers and heterozygous extensive/intermediate metabolizers had a significantly increased risk of recurrence compared with extensive metabolizers (poor metabolizers had a time to recurrence HR of 1.90, 95% CI, 1.10–3.28, whereas extensive/intermediate metabolizers had a time to recurrence adjusted hazard ratio [HR] of 1.40; 95% confidence interval [CI], 1.04–1.90) [122]. Patients with reduced CYP2D6 activity (heterozygous extensive/intermediate and poor metabolism) had worse event-free survival (HR, 1.33; 95% CI, 1.06–1.68) and disease-free survival (HR, 1.29; 95% CI, 1.03–1.61) compared to extensive metabolizers. The data from the Italian Tamoxifen Trial demonstrated a higher risk of disease relapse in women with the cytochrome P450 (CYP) 2D6 *4/*4 genotype [123]. Similar results were obtained by Goetz et al. [124], who observed that tamoxifen-treated women with the CYP2D6 *4/*4 genotype tend to have a greater risk of disease relapse and a lower incidence of hot flashes. Moreover, they also found that patients with a diminished metabolism had a significantly shorter time to recurrence (*p* = 0.034; adj. HR = 1.91; 95% CI 1.05–3.45) and worse relapse-free survival (RFS) (*p* = 0.017; adj. HR = 1.74; 1.10–2.74) compared to extensive metabolizers [125]. PM were indicated to display the most significant risk of breast cancer relapse (HR 3.12, *p* = 0.007) compared to EM. Teh et al. [126] reported an increased risk of recurrence and metastasis (OR 13.14; 95% CI 1.57–109.94; *p*  =  0.004) in Asian populations with CYP2D6*10/*10 and a heterozygous null allele (IM) compared with those with CYP2D6*1/*10 and *1/*1 genotypes. In contrast, another prospective study failed to find a relationship between *CYP2D6* variants and a pathological response or hot flashes; however, they observed a significant association between *CYP2D6* variants and Ki-67 response after preoperative tamoxifen therapy (*p* = 0.018) [127]. In turn, Günaldı et al. [128] reported correlations between the PM phenotype (*3/*4, *6/*6) and hyperplasia, as well as between the UM phenotype (3X*1/*1 duplication, 2X*1/*2) and atrophy in BC patients (*p*  =  0.019 for both cases). The presence of the CYP2D6*10/*10 allele was shown to decrease the disease-free survival (DFS), time to progression (TTP), and overall survival (OS) in East and Southeast Asia patients with breast cancer [129,130]. The efficacy of tamoxifen and its toxicity may also be affected by variations in genes other than *CYP2D6*. The phase II enzymes sulfotransferase family 1A member 1 (SULT1A1) and uridine 5′-diphospho-glucuronosyltransferase (UGTs) are also involved in the metabolism of tamoxifen and its metabolites. According to studies, polymorphisms with their genes may also contribute to differences in the concentration of circulating endoxifen, and thus to patients’ responses to treatment [19]. Human sulfotransferase 1A1 (SULT1A1) catalyses the sulfation of, among others, the active metabolite of tamoxifen, 4-hydroxytamoxifen (4-OH TAM). Functional polymorphisms within the *SULT* gene may not only alter enzymatic activity, thus affecting the therapeutic response, but also modify cancer susceptibility [131,132]. A functional polymorphism in exon 7 of the *SULT1A1* gene (SULT1A1*2) was found to be associated with an approximately twofold lower enzyme activity, and to be less thermostable than the common allele SULT1A1*1 [133]. Nowell et al. [133] observed that tamoxifen-treated patients who were homozygous for SULT1A1*2 (a low-activity allele) had an approximately three-times higher risk of death (HR = 2.9, 95% confidence interval [CI] = 1.1 to 7.6) compared to homozygous carriers of a common allele, or heterozygous carriers (SULT1A1*1/*2). The retrospective study of breast cancer patients treated with tamoxifen demonstrated that a high-activity genotype of a phase II enzyme UGT2B15 facilitating the elimination of active metabolite was associated with a higher risk of recurrence and poorer survival [134]. The combined analysis of *UGT2B15* and *SULT1A1* “risk” indicated that women carrying these two variant alleles had a markedly greater risk of recurrence and poorer survival compared with women with common alleles. Furthermore, a genetic variability in the UGT1A gene-encoding enzyme involved in the elimination of tamoxifen and its active metabolites has been suggested to influence tamoxifen therapy. UGT1A1*6 (A/A+A/G vs. G/G) (*p*  =  0.02) was found to decrease distant disease-free survival in Chinese patients [135].

Organic anion-transporting polypeptides (OATPs) are involved in the transport of exogenous and endogenous substances, including drugs (statins, methotrexate, Olmesartan, etc.); therefore, it is plausible that variations within their genes may affect the efficiency of some drug therapies [136,137]. Numerous studies have indicated that *OATP1B1* is highly polymorphic (at least 40 mutation have been identified so far). Among them, 521 T>C SNP in *OATP1B1* was found to have a reduced transport function, which translated into a higher concentration of drugs in the blood and better therapeutic effects due to the lower efficiency of the transport of drugs to hepatocytes [138]. In turn, the studies analysing the impact of another polymorphisms, i.e., 88A>G, in the same transport protein brought conflicting results. Some of them indicated an enhanced function of OATP1B1, while others failed to show such an effect [137]. This may be associated with the fact that *OATP1B1* polymorphisms show racial differences. There are hardly any studies of the impact of polymorphisms within *OATP1B1* on treatment outcomes. In vitro studies demonstrated that *OATP1B1* 388GG and 521CC reduced the activity of the OATP1B1 protein, limited its turnover capacity, and diminished the entrance of tamoxifen into MCF-7 cells, leading to the deteriorated efficacy of this drug in the treatment of breast cancer [139]. The in vitro analysis of the uptake of tamoxifen and its metabolites with the use of the overexpression lentivirus platform of wild-type and mutant-type (mutations at 388 and the 521 base) *OATP1B1* revealed that both tamoxifen and endoxifen could be taken up by the cells via OATP1B1; however, the presence of *OATP1B1* 521T/C polymorphism markedly inhibited the function of the transport protein. These results imply that *OATP1B1* 521T/C can hamper the effects of *OATP1B1* on tamoxifen and endoxifen in the cells.

### 3.4. 6-Mercaptopurine (6-MP)

The purine antimetabolite 6-Mercaptopurine (6-MP) is used in the treatment of leukaemia [55]. The mechanism of its antitumor action is based on the inhibition of the formation of the nucleotides that are required for the synthesis of DNA and RNA. The conversion of 6-MP into inactive metabolites is associated with the activity of thiopurine methyltransferase (TPMT), which catalyses its S-methylation. Therefore, functional genetic polymorphisms within the *TPMT* gene may significantly affect drug bioavailability and toxicity. A disturbed metabolic balance between the activation and inactivation of a prodrug, resulting for example from decreased TPMT activity, can lead to life-threatening bone marrow toxicity and myelosuppression [140]. So far, at least 24 genetic variants have been identified; however, only several appear to be clinically relevant [141,142]. The mode of inheritance of low- and high-activity TPMT is autosomally co-dominant. In the Caucasian population, 89% of individuals possess high (normal) enzyme activity (TPMT*1), 11% have intermediate activity, and 0.3% have a low activity [143,144,145]. The variant alleles TPMT*2–TPMT*24 show slightly-to-drastically reduced activities [142]. More than 80% of individuals with low TPMT activity are carriers of the following non-synonymous coding polymorphisms: TPMT*2 (G238C; Ala80Pro), TPMT*3A (G460A/A719G; Ala154 hr/Tyr240Cys), TPMT*3B (G460A; la154 hr) and TPMT*3C (A719G; Tyr240Cys), which are associated with alterations in the sequence of the encoded protein [141,145]. The presence of three alleles of *TPMT* (TPMT*2, TMPT*3A, and TPMT*3C) has been found to be responsible for nearly 95% of the observed cases of TPMT deficiency [146]. The activity of *TPMT* in TPMT*3C carriers is reduced two times, in TPMT*3B it is reduced nine times, and those with the TPMT*3A allele display negligible TPMT activity. Proteins encoded by all of the aforementioned alleles undergo rapid proteolytic degradation, which results in enzyme deficiency [147]. According to estimations, c.a. one in 300 individuals has a *TPMT* deficiency (an autosomal recessive trait). Such patients are characterized by a considerably reduced rate of 6-MP metabolism; thus, homozygous carriers of the TPMT*3A allele are at the greatest risk of developing life-threatening myelosuppression while they are treated with standard doses of thiopurines [148,149,150]. A study evaluating excessive toxicity in patients receiving mercaptopurine demonstrated the over-six-times overrepresentation of *TPMT* deficiency or heterozygosity among patients who developed hematopoietic toxicity from therapy containing thiopurines. These patients with bone marrow intolerance to 6-MP experienced more frequent hospitalization, more platelet transfusions, and more missed doses of chemotherapy. According to the authors, following appropriate dosage adjustments, *TPMT*-deficient and heterozygous patients can be administered thiopurines without eliciting acute dose-limiting toxicity [151]. Therefore, it seems that the analysis of genetic variations in the *TPMT* gene may enable the determination of a safe starting dose for 6-MP therapy [152]. Based on sound pharmacogenetic evidence, the FDA has decided to include information concerning the need for genotyping in the drug label for 6-MP [153]. The results of large cohort studies pointed to polymorphism within the nudix hydrolase 15 gene (*NUDT15*, also known as *MTH2*) as a vital factor determining 6-MP intolerance [154]. This nucleotide triphosphate diphosphatase converts oxidized GTP to its monophosphate form, which hampers the integration of the damaged purine nucleotides into DNA [155,156]. In turn, the excessive accumulation of tGTP/tdGTP results in extensive DNA damage and cytotoxicity [157]. It appears that variant rs116855232 is of high clinical importance. Carriers of the TT genotype could tolerate only around 10% of the dose tolerated by those with the CC genotype, while carriers of CT could tolerate 75% of said dose. The results of an immunochip-based assay demonstrated the relationship between the missense SNP 415C>T of the *NUDT15* gene (rs116855232) (resulting in a p.Arg139Cys change) and early leukopenia in thiopurine-treated patients [158]. The presence of the T allele raised the risk of leukopenia ~8 times compared to the C allele (*p* < 0.00001, OR = 7.86, 95% CI: 6.13, 10.08) [159]. Moreover, patients carrying the T allele tolerated a lower mean daily thiopurines dose (*p* < 0.00001). According to Moriyama et al. [160], the p.Arg139Cys change is associated with lower protein stability, which probably results from a loss of supportive intramolecular bonds and thus rapid proteasomal degradation in cells, rather than with decreased NUDT15 enzymatic activity. Another clinical trial enrolling children with acute lymphoblastic leukemia demonstrated that variants (p.Arg139Cys, p.Arg139His, p.Val18Ile and p.Val18_Val19insGlyVal) were associated with a 74.4–100% loss of nucleotide diphosphatase activity [160]. The presence of loss-of-function *NUDT15* diplotypes translated to thiopurine intolerance. Because NUDT15 inactivates thiopurine metabolites and reduces thiopurine cytotoxicity, patients carrying defective *NUDT15* alleles display excessive levels of thiopurine active metabolites and higher toxicity. The evidence of the relationship between *NUDT15* alleles and 6-MP intolerance is considerable; it is recommended to perform genetic tests for NUDT15 before the start of thiopurine therapy [161]. Furthermore, TPMT testing is recommended. Patients with *NUDT15* and *TMPT* genetic variants require the adjustment of the drug dose. According to CPIC recommendations, normal metabolizers (e.g., *NUDT15* *1/*1, MP 75 mg/m2/day in ALL) should be administered with the normal starting dose, while intermediate metabolizers (e.g., *NUDT15* *1/*3, MP 30–80% of the normal starting dose) and poor metabolizers (e.g., *NUDT15* *3/*3; MP 10 mg/m2/day in ALL) should receive a reduced dose [161]. Genotype-tailored, individualized dosing holds the promise of the minimization of adverse drug reactions.

### 3.5. Sunitinib

Sunitinib malate, a multitarget tyrosine kinase inhibitor, is a well-established chemotherapeutic for the treatment of metastatic renal cell carcinoma (mRCCs), gastrointestinal stromal tumours (GISTs), metastatic breast cancer, and other types of solid tumours [162]. This drug has been approved in the United States and the European Union for the treatment of advanced renal cell carcinoma and imatinib-resistant or imatinib-intolerant gastrointestinal stromal tumours [163]. Sunitinib blocks the receptors of vascular endothelial growth factor (VEGFR-1, 2, and 3), the platelet-derived growth factor receptors α and β (PDGFR-α and PDGFR-β), the stem cell factor receptor (KIT), and Fms-like tyrosine kinase-3 receptor (FLT3), as well as the glial cell line–derived neurotrophic factor receptor [163,164,165]. CYP3A4 converts sunitinib into its active N-desethyl metabolite (SU12662), and further metabolizes it into inactive metabolites [162]. The clinical benefits from sunitinib therapy may depend on inter-individual variations in drug absorption, metabolism, distribution, and excretion [166]. The results of studies have indicated that a higher exposure to sunitinib is associated with improved survival, but also with an greater risk for adverse events [163,167]. Clinical evidence indicates that the individual response to sunitinib is highly variable due to extensive differences in the plasma concentration following standard dosage regimens. Phase I studies in patients with advanced solid tumours determined the maximum tolerated dose (MTD, the maximum dose with less than 33% incidence of dose-limiting toxicity) of sunitinib, which is 50 mg daily [167,168]. Some patients administered with the recommended dose show no response, while others experience severe toxicity and require dose limitation (~32–46%) or treatment discontinuation (38%). The most frequent toxicities involve hand–foot skin reactions and haematological toxicities [169]. However, there are no established markers which could enable the prediction of the efficacy and toxicity [170]. Some studies have suggested the importance of the mutant CYP3A5*3 allele, which is associated with the defective CYP3A5 enzyme [171]. The presence of a defective CYP3A5 enzyme may result in the accumulation of the parent drug [172]. This is especially important in the Asian population, in which race and low body weight were found to reduce sunitinib clearance, exacerbating sunitinib toxicity [163]. However, Numakura et al. [170]—who analysed the impact of single-nucleotide polymorphisms (SNPs) in genes related to sunitinib pharmacokinetics (the transport proteins ATP-binding cassette *ABCB1*: rs1045642, rs1128503, rs2032582, and rs7779562; and *ABCG2*: rs2231142, and *CYP3A4* (rs35599367) and *CYP3A5* (rs776746)) on clinical outcomes in Japanese patients with mRCC—failed to find any associations between studied SNPs and dose reduction, progression-free survival, overall survival, and the best objective response. Furthermore, Teo et al. [169] also suggested that the presence of variations in the *CYP3A5* may not affect the metabolism of this drug due to the redundancy between CYP3A5 and CYP3A4 enzymes. It appears that the relative metabolizing capacity of CYP3A4 for sunitinib may adequately compensate for any variability in the CYP3A5 enzyme. Moreover, sunitinib seems to be a better substrate for CYP3A4 compared with CYP3A5 [173]. In contrast, one of studies demonstrated that the presence of CYP3A5*1 was associated with dose reductions due to toxicity (odds ratio: 2.0; 95% CI, 1.0–4.0, *p* = 0.039) [174]. Diekstra et al. [175] observed that, in a Caucasian population, the clearance of sunitinib can be affected by the CYP3A4*22 polymorphism. However, this polymorphism was not detected in Asians. The results of all of the aforementioned studies confirm the importance of CYP3A4 in sunitinib metabolism. Furthermore, polymorphisms within genes encoding efflux transporters and drug targets may affect the efficacy of sunitinib, as well as sunitinib-induced toxicities [166]. SNPs in VEGFR-2 have been found to modulate sunitinib activity. For example, *VEGFR*-2 1718T/A was associated with the lower overall survival of sunitinib-treated patients [166]. Another study revealed that the *VEGFR*-2 (T allele in 1191 C/T) was associated with the occurrence of any toxicity > grade 2, while *NR1I3* (the absence of a CAG copy in the haplotype) and *ABCB1* (the presence of a TTT copy) polymorphisms enhanced the risk of leukocytopenia and hand–foot syndrome, respectively [176].

Sunitinib is a substrate of ABCB1 and ABCG2 [177]. SNPs within *ABCB1* (a TCG copy) and *ABCG2* (e.g., 421C/A) may influence sunitinib absorption and excretion. The presence of both of the aforementioned polymorphisms was associated with a better outcome [166]. The improved outcome may result from the diminished efflux transport of sunitinib into the gastrointestinal lumen and bile, and the consequent enhanced systemic exposure of sunitinib. The carriers of the *ABCG2* rs2231142 AA genotype were found to be more likely to develop thrombocytopenia, neutropenia, and hand–foot syndrome [178]. This SNP is located within the ATP-binding cassette domain; as such, it can modulate the ATP-binding activity of the ABCG2 protein [179]. The presence of the A allele was found to decrease such activity and diminish transport capability, leading to drug accumulation and the reduced efflux velocity of the drug [180]. In turn, Chu et al. [181] marked the relationship between ABCB1 1236T (OR = 0.3), *ABCB1* 3435T (OR = 0.1), *ABCB1* 2677T (OR = 0.4), and *ABCG2* 421A (OR = 0.3) alleles and the *ABCB1* 3435, 1236, 2677 TTT haplotype (OR = 0.1) with neutropenia in Asian mRCC patients. The *ABCB1* 3435, 1236, 2677 TTT haplotype conferred primary resistance (OR = 0.1, *p* = 0.004) as well as inferior survival (progression-free: hazard ratio [HR] = 5.5, *p* = 0.001; overall: HR = 5.0, *p* = 0.005). Moreover, Beuselinck et al. [182] revealed the link between SNP rs1128503 in *ABCB1* and progression-free survival (PFS) and overall survival (OS) (*p* = 0.027 and *p* = 0.025) in sunitinib-treated metastatic clear-cell RCC. However, Garcia-Donas et al. [183] failed to observe such an association. The carriers of variants rs1128503 and rs2032582 in *ABCB1* display an enhanced clearance of sunitinib and its active metabolite (SU12662), which results in a lower exposure to the drug [169,174]. A recent meta-analysis demonstrated that, in carriers of the T allele (*ABCB1* rs1128503), the risk of sunitinib-induced hypertension was considerably reduced compared to those with the C allele; however, progression-free survival was shorter in this group of Asian and Caucasian patients [177]. Based on the results of studies, it seems quite reasonable to genotype for *ABCG2* rs2231142, and *ABCB1* rs1128503 and rs2032582 polymorphisms in order to adjust the drug dose and reduce the risk of sunitinib-induced thrombocytopenia and hand-to-foot syndrome in Asians in whom the prevalence of SNPs within ABCG2 is high.

### 3.6. Mitotane

A highly lipophilic compound, mitotane, is the most effective agent in the post-operative treatment of adrenocortical carcinoma which has been approved by the US FDA and the European Medicines Agency [184,185,186]. Despite the fact that it has been used for a long time, many pharmacological aspects—including its activation and pharmacodynamics—require further studies, as the knowledge in this field is sparse. It has been found that mitotane blocks sterol-O-acyl transferase 1, which results in disturbed steroidogenesis and lipid-induced endoplasmic reticulum stress [187]. According to studies, high oral daily doses (1–6 g/day) are needed in order to reach therapeutic concentrations [187]. In adults, an initial dose of 2–3 g/day is usually used, and it should be carefully increased in order to attain a therapeutic range of plasma concentration of 14 and 20 mg/L. Mitotane has a narrow therapeutic index [187]. Moreover, the management of a patient treated with this drug is complicated due to the very long elimination half-life related to its strong drug diffusion in adipose tissues and organs, and drug interaction via the stimulation of metabolizing enzymes. Such high doses require therapeutic drug monitoring (TDM) [186]. The problems with the reliable prediction of appropriate mitotane plasma concentrations may, on the one hand, translate to delayed tumour treatment, and on the other may result in drug toxicity. Poor mitotane tolerability is associated with frequent reductions of the dose, or even the suspension of the therapy as a result of a high rate of side effects. Both the efficacy and toxicity of mitotane are related to its plasma concentration [187]. According to the estimations, only half of the patients treated with a high-dose regimen for 3 months reached the target. This observation suggests a high inter-individual variability in mitotane pharmacokinetics, and indicates the need for individualized treatment [188]. Presently, the dosage titration is basically expert-based. Therefore, a tool assisting the determination of the mitotane concentration and enabling the selection of an optimized treatment regimen for individual patients is required.

Mitotane strongly stimulates CYP3A4; at the same time, this enzyme is involved in mitotane metabolism. According to Arshad et al. [187], the increase in the clearance of mitotane during treatment could be modelled by a linear enzyme autoinduction process. Genetic variability in the *CYP3A4* gene in the Caucasian population is limited; therefore, it seems that polymorphisms in the second enzyme metabolizing this drug (CYP2B6) may be of importance [189,190]. The retrospective analysis of patients with adrenocortical carcinoma on postoperative adjunctive mitotane demonstrated that SNP in *CYP2B6* (rs3745274) affected mitotane concentrations after three months of treatment [191]. Patients carrying the GT/TT genotype had higher mitotane plasma concentrations compared to patients with GG at 3 months (14.80 vs. 8.01 μg/mL; *p* = 0.008) and 6 months (17.70 vs. 9.75 μg/mL; *p* = 0.015). However, this difference in mitotane levels was no longer statistically significant after 9 months. In turn, Mornar et al. [192] suggested that *CYP2C9* variability may also affect mitotane concentrations. In their study, a high mitotane level was observed in CYP2C9 intermediate metabolizers. According to these authors, the mitotane dose should be adjusted on the basis of the determination of the following three SNP: CYP2C19*2 (rs4244285), *SLCO1B3* 699A/G (rs7311358) and *SLCO1B1* 571T/C (rs4149057); however, further confirmation of the obtained results is required [192]. CYP2C19*2, which is a non-functioning variant diminishing the activity of CYP2C19, was found to be in 100% linkage disequilibrium with *CYP2C18* 1154C/T (rs2281891) [155]. The sterol O-acyltransferase enzyme (SOAT1) has been found to be the vital molecular target of mitotane [193]. The association between its expression and the outcome of adjuvant mitotane treatment has been shown in some studies [193,194]. Some other studies failed to observe that SOAT1 expression could predict the treatment response to mitotane [193,195]. Moreover, the expression level of SOAT1 was found not to be associated with the recurrence-free survival, progression-free survival, or disease-specific survival of adrenocortical cancer patients treated with mitotane [196].

### 3.7. Imatinib

Imatinib is a tyrosine kinase inhibitor which is used for the treatment of chronic myeloid leukaemia (CML) [197]. CML is a myeloproliferative disorder resulting from the reciprocal translocation between chromosomes 9 and 22, leading to the fusion of the Abelson murine leukemia viral oncogene homolog 1 (*ABL1*) gene from chromosome 9 with the breakpoint cluster region (*BCR*) gene on chromosome 22 [198]. As a consequence, the BCR-ABL1 fusion gene (Philadelphia chromosome (Ph)) is formed [197]. The protein product of this gene is a constitutively active tyrosine kinase activating many oncogenic signalling pathways, including RAS/RAF/MEK/ERK, PI3K/AKT/mTOR, and JAK/STAT [197,199]. The introduction of imatinib as a frontline therapy for chronic-phase CML resulted in a significant increase in the 5-year survival rates (from 31% in early the 1990s to 66% in 2012) [197]. More recent clinical trials have indicated even higher survival rates, with a 10-year overall survival as high as 83.3% [200]. Imatinib mesylate (IM) is a highly efficient first-line therapy for the treatment of chronic myeloid leukaemia; however, resistance to this therapy has emerged as a serious clinical problem [201]. CYP3A4 is the chief enzyme involved in the first-pass metabolism of imatinib. However, other enzymes (CYP3A5, CYP2C8, and CYP2D6), but to a lesser degree, are also involved in this process. Following its administration, imatinib is converted by the CYP3A enzymes into pharmacologically active N-desmethyl imatinib, which shows a 3–4 times lower cytotoxicity than imatinib [202,203]. A pilot study on in vivo CYP3A activity demonstrated a higher in vivo CYP3A activity in patients who achieved a complete molecular response (Mann–Whitney U-test, *p* = 0.013; median quinine metabolic ratio = 10.1) compared to those who achieved a partial molecular response (median = 15.9) [204]. However, some more recent data suggest that the previous studies underestimated the role of other CYP450 enzymes. Filppula et al. [205] demonstrated that the role of CYP3A4 is crucial during the first period after the initiation of imatinib treatment (hepatic clearance by CYP2C8 and CYP3A4 accounts for 40 and 60%, respectively); however, during long-term treatment with imatinib at 400 mg once or twice daily, the predominant role of CYP3A4 may be taken over by CYP2C8 (CYP2C8: 65–75% hepatic elimination; CYP3A4: 25–35% hepatic elimination). This finding may be associated with the dose- and time-dependent auto-inactivation of CYP3A4 by imatinib [205]. In the course of multiple dosings, both the polymorphisms and drug interactions affecting the function of CYP2C8 may be the source of inter-individual variation in response to imatinib, as well as the occurrence of side-effects. The presence of CYP3A5*3 was found to be associated with the appearance of a cryptic splice site which results in a premature stop codon and the subsequent absence of functional CYP3A5 protein [206]. The frequency of this allele is quite low in Caucasians (5–10%) but very high in Africans and African-Americans (60%). Data concerning the influence of CYP3A5*3 on drug pharmacokinetics, efficacy, and toxicity seems to be incomplete due to the fact that most drugs metabolized by CYP3A5 also undergo conversion by CYP3A4 [23]. Maddin et al. [207] demonstrated that CML patients with at least a CYP3A5*1 polymorphic allele tended to express greater amounts of CYP3A5. Moreover, they observed a considerably lower risk of developing resistance against imatinib in the heterozygous (*1/*3) and homozygous (*3/*3) variant carriers, which may suggest that this allele exerts protective effects. Furthermore, the Canadian study implied that carriers of the CYP3A5*1/*1 genotype showed a higher risk of developing resistance to IM [208]. A study of Nigerian CML patients assessing the impact of CYP3A5*3 polymorphisms on imatinib metabolism revealed that the GG genotype was associated with considerably greater trough plasma concentrations; however, no correlation between this level and the clinical outcomes was noted [209]. Individuals with at least one CYP3A5*1 allele have a high concentration of CYP3A5; therefore, it appears that heterozygous or homozygous carriers of CYP3A5*1 should show a high rate of clearance and the lowest oral bioavailability of CYP3A substrates; therefore, these patients may not benefit from the standard dose of a drug [207]. In turn, homozygous patients with the CYP3A5*3 genotype may experience reduced enzyme activity, leading to the limited clearance and high bioavailability of the drug, and the subsequent better response to IM, but also a possibly increased risk of adverse events. Another study which assessed the functional impact of *CYP2B6* 15631G/T polymorphism on the response of imatinib in CML patients demonstrated the relationship between a higher hematologic response and the presence of the 15631GG/TT genotype compared to 15631GT (36.8 vs. 13.8%; χ^2^ = 3.542, *p* = 0.063) [210]. However, the complete cytogenetic response was better in patients carrying the 15631GG/GT genotype when compared with 15631TT (χ^2^ = 3.298, *p* = 0.024), while the primary cytogenetic resistance was greater in those with the 15631GG/TT genotype when compared with 15631GT carriers (52.6 vs. 17.2%; χ^2^ = 6.692, *p* = 0.010). The results of this study indicate the better response of patients with 15631GG alleles; however, this group was also more susceptible to side effects (*p* = 0.004). 

These findings provide initial evidence that the determination of polymorphisms within P450 enzymes could help to predict the therapeutic response to imatinib. However, the observed associations should be treated with caution due to the fact that the reported variants may be population-specific; therefore, such relationships must be confirmed in larger CML cohorts in order to assess the clinical relevance.

The intracellular levels of tyrosine kinases such as imatinib depend on their influx and efflux involving transmembrane transporter proteins [197]. This process is associated with the efficiency of BCR-ABL1 inhibition. Therefore, polymorphisms within genes encoding proteins responsible for drug efflux, including *ABCB1* (also called *MDR1* or *P-GP*) and *ABCG2* (also known as *BCRP2*), may modulate the effects of imatinib treatment. Some studies have demonstrated increased ABCB1 expression in the advanced stages of CML, as well as the relationship between higher ABCB1 expression and a lower rate of imatinib resistance [211,212,213]. The results of in vitro studies pointed to a rise in ABCB1-mediated drug efflux as a plausible mechanism of resistance to imatinib [214]. The study of the three most frequent SNSs (1236T/C, 2677G>T/A and 3435C/T) in patients with CML or gastrointestinal stromal tumours (GIST) revealed considerably greater imatinib clearance in carriers of the TT genotype at all three loci [215]. Some studies have demonstrated significantly higher rates of major molecular response (MMR) to imatinib in patients with 1236TT or 2677TT/TA, while others have reported reduced rates of MMR and complete molecular response [216,217]. In vitro studies have not brought unequivocal answers as to whether the aforementioned polymorphisms also play a role in the modulation of the imatinib response [218,219]. In one issue on which the scientists agree, the level of ABCB1 expression strongly correlates with imatinib responsiveness [211,213]. Furthermore, the expression of ABCG2 appears to be involved in the mediation of TKI resistance. A study of two variants, *ABCG2* 34G/A and 421C/A, in Malaysian patients with CML found a considerably better response to imatinib in carriers of diplotype A34A421 [220]. In turn, a meta-analysis of 14 studies and nearly 2200 patients indicated a markedly higher MMR and complete cytogenetic remissions in CML patients carrying the 421A variant [221]. Moreover, the polymorphisms within the solute carrier family 22 member SLC22A1 (or organic cation transporters or OCT1) may modulate treatment effects because they control the active intracellular uptake of TKIs [197]. Again, the results of studies concerning the impact of variations within OCT1 on the imatinib response are conflicting. Some studies confirmed that SLC22A1 expression correlated with the imatinib response over time [222,223]. In Asian patients with CML, rs3798168, rs628031, and IVS7+850C>T polymorphisms were found to be considerably associated with imatinib clearance [224]. In turn, a study of Italian patients with CML demonstrated a relationship between SLC22A1 480C/G SNP and imatinib clearance [225]. The presence of at least one G allele was suggested to be associated with significantly reduced imatinib clearance [225]. Another study found a considerably higher frequency of heterozygous (CG) and homozygous variant (GG) genotypes of SLC22A1 C480G in the IM-resistant group compared with the IM good-response group [226]. Moreover, the authors suggested that carriers of 1222AA—both 8-bp insertion and 3-bp deletion—and M420del alleles had an increased risk of developing resistance towards IM treatment. The *SLC22A1* variants L160F (rs683369, C480G) and *M408V* (rs628031, A1222G) are most frequently associated, in the available studies, with the imatinib response [227]. The results of some studies have suggested an increased risk of imatinib resistance and reduced event-free survival in homozygous carriers of the L160F variant, while others failed to observe such an association [208,225,228,229,230]. Homozygous carriers of the CC genotype had a considerably lower steady-state imatinib plasma concentration, which suggests the impact of the L160F variant on imatinib pharmacokinetics [227]. Grinfeld et al. [231] found an interaction between the M420del variant and rs113569197 (TGGTAAGT insertion [8+]). In their study, patients lacking both the 8+ and M420del variants had a superior overall outcome. In turn, Singh et al. [224] identified the sub-haplotypic region of *OCT1* involving IV6-878C/A (rs3798168), M408V and IVS7+850C/T, which influenced imatinib clearance. 

Table 1 comprises the summary of studies concerning the impact of polymorphisms within genes encoding enzymes involved in the metabolism of drugs on therapeutic effects

## 4. Conclusions

Anticancer drugs usually have a very narrow therapeutic index; therefore, it is very important to use appropriate doses in order to achieve the maximum benefits without putting the patient at risk of life-threatening toxicities. However, the adjustment of the appropriate dose is not so easy due to the inheritance of specific polymorphisms in the genes encoding the target proteins and drug-metabolizing enzymes. This review presented just a few examples of such polymorphisms and their impact on the response to therapy. It appears that the characterization of all of the genetic polymorphisms present in humans and the understanding of their role in clinical endpoints would enable the development of clinical practice strategies based on accurate genotype testing, and would facilitate the rational selection of cancer drug(s) and the adjustment of the dosage for the individual patient. Furthermore, the knowledge of drug–drug interactions is of high importance in this field. Such an approach would allow for the optimization of treatments. 

## Figures and Tables

**Table 1:** The summary of studies concerning the impact of polymorphisms within genes encoding enzymes involved in the metabolism of drugs on therapeutic effects.

Name of Drug	Enzyme	Polymorphisms	Result of the Presence of Polymorphism	Clinical Translation	Ref
5-fluorouracil (5-FU)	Dihydropyrimidine dehydrogenase (DPD)	1905+1G/A & 1679T/G (strong impact) 2846A/T & 1129–5923C/G (moderate impact)	IM: (1 normal function allele + 1 no function allele or 1 decreased function allele, or 2 decreased function alleles) Decreased DPD activity	Increased risk for severe or even fatal drug toxicity when treated with fluoropyrimidine drugs Solution: Reduction of starting dose followed by titration of dose based on toxicity or therapeutic drug monitoring	[30]
		1905+1G/A & 1679T/G (strong impact) 2846A/T & 1129–5923C/G (moderate impact)	PM: (2 no function alleles or 1 no function + 1 decreased function) Complete DPD deficiency	Increased risk for severe or even fatal drug toxicity when treated with fluoropyrimidine drugs. Solution: Avoid use of 5-FU or 5-FU prodrug-based regimens. If alternative agents are contraindicated- use 5-FU at a strongly reduced dosed with early therapeutic drug monitoring.	[30]
		IVS14 + 1G/A or 2846A/T	Decreased DPD activity	Early grade 3 to 4 toxicity Solution: Treatment should be quickly stopped, or safely continued with an individual dose adjustment.	[38]
		Homozygotes - IVS14 + 1G/A allele are	Complete lack of DPD activity	5-FU-related toxicities can be life-threatening or sometimes even fatal	[40]
Irinotecan	UDP glucuronosyltransferase family 1 member A1 (UGT1A1)	−3156G/A and additional TA repeat in the TATA sequence of the UGT1A1 promoter, ((TA)7TAA, instead of (TA)6TAA) (UGT1A1*28)	Gene transcriptional efficiency is inversely correlated to the number of TA repeats in the TATA box	Greater risk of grade 4 neutropenia in patients with the TA indel 7/7 genotype (relative risk: 9.3 (95% CI, 2.4 to 36.4)) compared with 6/7 and 6/6 (*p* = 0.001) TA indel genotype was significantly associated with the absolute neutrophil count nadir (7/7 < 6/7 < 6/6, *p* = 0.02).	[71]
		additional TA repeat in the TATA sequence of the UGT1A1 promoter, ((TA)7TAA, instead of (TA)6TAA) (UGT1A1*28)	(TA)7TAA: significantly lower SN-38 glucuronidation rates compared (TA)6TAA) (*p* = 0.001)	(TA)7TAA heterozygous carriers: more severe grades of diarrhoea (4 grade) and neutropenia (TA)7TAA homozygous carriers: grade 3 diarrhoea/grade 4 neutropenia, Conclusion: screening for UGT1A1*28 polymorphism may identify patients with greater susceptibility to irinotecan induced gastrointestinal and bone marrow toxicity.	[72]
		UGT1A1*6 UGT1A7*3 UGT1A9-118(T)9/9	UGT1A variants: lower enzyme activity	UGT1A1*6/*6: higher incidence of severe neutropenia, lower tumour response, shorter progression-free and overall survival compared with other genotypes. UGT1A7*3/*3: lower drug response rate (*p* = 0.034) UGT1A9-118(T)9/9 or UGT1A7*3/*3: high incidence of grade 3 diarrhoea (*p* = 0.037 and *p* = 0.28, respectively) Conclusions: UGT1A1*6 and/or UGT1A9*22 genotypes might be important for predicting severe toxicity and treatment outcome after irinotecan-based chemotherapy	[77]
		UGT1A1*28 (seven repeats (TA_7_))	Reduced efficiency of transcription of the UGT1A1 gene	Recommended irinotecan dose of 180 mg/m^2^ is considerably lower than the dose that can be tolerated for patients with the UGT1A1 *1/*1 and *1/*28 genotypes. Maximum tolerable dose for:	[89]
				- high-risk UGT1A1 *28/*28 genotype is 30% lower than the standard dose of 180 mg/m^2^. - *1/*1 genotype: 450 mg/m^2^ - *1/*28 genotype: 390 mg/m^2^ - *28/*28 150 mg/m^2^	
Tamoxifen	CYP2D6	CYP2D6*1, *2, *3,*4, *5, *6, *7 *9, *10, *16, *16, *1C (T1957C), *2B (additional C2558T), and *4E (additional C2938T)	EM phenotype: CYP2D6*1 allele IM phenotype: slightly (CYP2D6*2) or moderately (*9 and *10) reduced activity PM phenotype: complete enzyme deficiency (*4; *3 and *5; *6; *7, *15, and *16).	Poor metabolizers: substantially lower doses would be optimal for this group	[117]
	CYP2D6	CYP2D6*3, *4, *5, *10 and *41 alleles	PM: lack of active enzyme function (homozygous or compound heterozygous for CYP2D6*3, *4, or *5 alleles) IM: reduced enzyme activity (*10 and *41 alleles either homozygous or in combination with a PM allele) EM: normal enzyme function (absence of PM and IM alleles) UM: high enzyme activity (duplicated gene copies without a PM or IM allele)	Significantly increased risk of recurrence in heterozygous EM/IM compared with EM (time to recurrence adjusted HR, 1.40; 95% CI, 1.04–1.90) and PM (time to recurrence HR, 1.90; 95% CI, 1.10–3.28). Decreased CYP2D6 activity was associated with worse event-free survival (HR, 1.33; 95% CI, 1.06–1.68) and disease-free survival (HR, 1.29; 95% CI, 1.03–1.61).	[122]
	CYP2D6	P450 (CYP)2D6 (*4 and *6) and CYP3A5 (*3) genotype	CYP2D6 *4/*4: PM phenotype	CYP2D6 *4/*4 genotype: worse relapse-free time (RF-time; *p* = 0.023) and disease-free survival (DFS; *p* = 0.012) CYP2D6 *4/*4 genotype: lower incidence of hot flashes The CYP3A5*3 variant: no impact on any of aforementioned clinical outcomes.	[124]
	CYP2D6	CYP2D6*10, CYP2D6*4, CYP2D6*5, CYP2D6*14	CYP2D6*10: reduced enzyme activity in IM CYP2D6*4, CYP2D6*5 and CYP2D6*14: null alleles encoding no enzyme at all	CYP2D6*10/*10 and heterozygous null allele (IM): higher risks of recurrence and metastasis (OR 13.14; 95% CI 1.57–109.94; *p* = 0.004) compared with CYP2D6*1/*1 and *1/*10 genotypes.	[126]
	Sulfotransferase 1A1 (SULT1A1)	SULT1A1*1 & SULT1A1*2	SULT1A1*2: enzyme with approximately twofold lower activity and less thermostable compared to SULT1A1*1	SULT1A1*2 homozygotes: ~3 times higher risk of death (HR = 2.9, 95%, CI = 1.1 to 7.6) compared to SULT1A1*1 homozygotes or SULT1A1*1/*2 heterozygotes.	[133]
	SULT1A1 and UDP-glucuronosyltransferase isoform 2B15 (UGT2B15)	SULT1A1*1 & SULT1A1*2, Asp85Tyr(UGT2B15*1/*2)	SULT1A1*2: decreased catalytic activity Asp85Tyr(UGT2B15*1/*2): increased velocity of reaction	UGT2B15*2 high activity genotypes: increased risk of recurrence and poorer survival. Combination of UGT2B15 and SULT1A1 ‘at-risk’ alleles: significantly greater risk of recurrence and poorer survival than those with common alleles.	[134]
6-Mercaptopurine (6-MP)	Thiopurine S-methyltransferase (TPMT)	TPMT*2, *3A, *3B, and *3C alleles	TPMT enzyme deficiency	TPMT-deficient patients experience more frequent hospitalization, more platelet transfusions, and more missed doses of chemotherapy.0 Serious side-effects: hematologic toxicity (>90% of patients) Following appropriate dosage adjustments, TPMT-deficient and heterozygous patients can be treated with thiopurines, without acute dose-limiting toxicity	[151]
Sunitinib	CYP3A4, CYP3A5	CYP3A4 (rs35599367) and CYP3A5 (rs776746)		No significant association between the genotypes of each SNP and time to dose reduction, progression-free survival, overall survival, and best objective response.	[170]
	CYP3A4 or CYP3A5	CYP3A5*1/*1, CYP3A5*3/*3		Sunitinib activated midazolam 1′-hydroxylation by CYP3A5 but inhibited that by CYP3A4. Unexpected drug interactions involving sorafenib and sunitinib might occur via heterotropic cooperativity of CYP3A5.	[173]
	CYP3A5	CYP3A5*1/*1, CYP3A5*3/*3		CYP3A5*1: need for dose reductions (OR: 2.0; 95% CI, 1.0–4.0, *p* = 0.039).	[174]
	CYP3A4, CYP3A5		SNPs in CYP3A4, CYP3A5, affected the clearance of both sunitinib and SU12662.	CYP3A4*22 was eliminated with an effect size of −22.5% on clearance	[175]
Mitotane	CYP2B6	G/T (rs3745274)	Affects mitotane metabolism	Significant correlation between CYP2B6 SNP and mitotane plasma levels (after 3 months) (*p* = 0.003). Patients with the GT/TT genotype: higher mitotane plasma concentrations after 3 months of treatment compared with patients with GG, the wild-type genotype [14.80 μg/mL (10.50–18.08) vs. 8.01 μg/mL (6.37.6–10.61); *p* = 0.008]	[191]
	CYP2C9 SLCO1B1 SLCO1B3	CYP2C19*2 (rs4244285), SLCO1B3 699A/G (rs7311358) and SLCO1B1 571T/C (rs4149057)	CYP2C19*2: a non-functioning variant diminishing the activity of CYP2C19	CYP2C9 IM: high mitotane level. Suggestion: the adjustment of mitotane dose based on the determination of three SNP: rs4244285, rs7311358 and rs4149057	[192]
Imatinib	CYP3A4 and CYP3A5	CYP3A5*3 (6986A/G) and CYP3A4*18 (878T/C)	Alter the enzyme activity of IM and may affect its response CYP3A5*1 polymorphic allele tended to express greater amounts of CYP3A5	Carriers of heterozygous (AG) and homozygous variant (GG) of CYP3A5*3: significantly lower risk of acquiring resistance with OR 0.171; 95% CI: 0.090–0.324, *p* < 0.001 and OR 0.257; 95% CI: 0.126–0.525, *p* < 0.001, respectively. Non-significantly lower risk of acquiring resistance toward IM in heterozygous carriers of TC genotype of CYP3A4*18 (OR 0.648; 95% CI: 0.277–1.515) (*p* = 0.316).	[207]
	CYP2B6	15631G/T	Decreases enzymatic activity of CYP2B6 in liver	15631GG/TT genotype: higher hematologic response loss compared with 15631GT (36.8 vs. 13.8%; X (2) = 3.542, *p* = 0.063). 15631GG/GT genotype: higher complete cytogenetic response compared with 15631TT χ^2^ = 3.298, *p* = 0.024). 15631GG/TT genotype: higher primary cytogenetic resistance compared with 15631GT carriers (52.6 vs. 17.2%; χ^2^= 6.692, *p* = 0.010). 15631GG genotypes: more frequent side effects compared with GT/TT carriers (36 vs. 13.8 %; χ^2^ = 8.3	[210]

## Data Availability

Not applicable.
